# Self-harming behavior linked to earlier onset of cardiovascular disease in severe mental disorders

**DOI:** 10.1192/j.eurpsy.2025.10106

**Published:** 2025-09-15

**Authors:** Synve Hoffart Lunding, Isabel Viola Kreis, Linn Rødevand, Monica Aas, Maren Caroline Frogner Werner, Ingrid Torp Johansen, Monica Bettina Elkjær Greenwood Ormerod, Elina Reponen, Gabriela Hjell, Petter Andreas Ringen, Trine Vik Lagerberg, Ingrid Melle, Ole Andreas Andreassen, Carmen Simonsen, Torill Ueland, Nils Eiel Steen

**Affiliations:** 1 Division of Mental Health and Addiction, Oslo University Hospital, Oslo, Norway; 2 Institute of Clinical Medicine, University of Oslo, Oslo, Norway; 3 Social, Genetic and Developmental Psychiatry Centre, Institute of Psychiatry, Psychology and Neuroscience, King’s College London, London, UK; 4 Department of Psychosis Studies, Institute of Psychiatry, Psychology & Neuroscience, King’s College London, London, UK; 5 Department of Research and Innovation, Division of Clinical Neuroscience, Oslo University Hospital, Oslo, Norway; 6 Department of Psychiatry, Ostfold Hospital, Graalum, Norway; 7 Department of Psychology, University of Oslo, Oslo, Norway; 8 Division of Mental Health and Substance Abuse, Diakonhjemmet Hospital, Oslo, Norway

**Keywords:** attempted suicide, bipolar disorders, cardiovascular disease risk, registry data, schizophrenia, self-harm

## Abstract

**Background:**

People with severe mental disorders (SMDs) have about 15 years shorter life expectancy than the general population. Cardiovascular disease (CVD) is among the leading causes of premature mortality and shares genetic underpinnings with SMDs. We investigated the link between clinical traits in SMDs and time to the first CVD diagnosis.

**Methods:**

The study included 1,627 well-characterized participants with schizophrenia spectrum (SCZ, *N* = 998) and bipolar spectrum disorders (BDs, *N* = 629), and a reference group of 1,201 healthy controls. CVD diagnoses were obtained from two Norwegian national registries (covering both primary and specialist health care) for the period of 2006–2020. Applying Cox proportional hazard models, we investigated associations between SMD clinical traits and time to first CVD diagnosis in SMD participants, adjusting for age, sex, diagnosis, and tobacco use.

**Results:**

Among individuals with SMD, recurring self-harming behavior (SHB) was associated with a shorter time to first CVD diagnosis (*p* = .029) relative to those without SHB. In the subgroup with SHB and a history of attempted suicide(s), more suicide attempts were associated with shorter time to first CVD diagnosis (*p* = .041). Significant associations of time to first CVD diagnosis with age at SMD onset and comorbid substance use disorder were not demonstrated.

**Conclusions:**

SHB and a history of suicide attempts in individuals with SMD seem to be associated with earlier CVD onset, and may improve the prediction of CVD, in addition to standard cardiovascular risk factors.

## Introduction

Severe mental disorders (SMDs), including schizophrenia spectrum (SCZ) and bipolar spectrum disorders (BDs), significantly impair quality of life [1] and functional abilities [2]. An excessive mortality of two to three times compared to the general population, translating into a shortened life expectancy by about 15 years [3, 4], adds to the burden. Along with high suicide rates [5], cardiovascular disease (CVD) remains a major cause of excess mortality in this population [6–10].

The elevated CVD risk in SMD is evident as early as young adulthood. However, recognition of this risk is often delayed [8, 11], and patients frequently receive inadequate somatic care [8, 12, 13]. Besides the metabolic side effects of antipsychotics [14, 15], SMD is associated with genetic variants linked to body mass index [16], lipid metabolism, and blood pressure regulation [17]. Shared genetics might also underlie CVD lifestyle risk factors, such as smoking [17], as well as personality and behavior-related traits linked to CVD [18–22]. Moreover, SMD genetic risk has been associated with specific clinical characteristics [23–25]. These underlying mechanisms indicate that clinical and behavior-related traits of SMD might inform CVD risk assessments, aiding early identification of individuals prone to CVD within mental health care settings.

While there are few studies on SMD clinical characteristics and CVD [26], the evidence suggests trait-like illness characteristics, such as duration of illness and number of hospitalizations, rather than state measures, to be associated with higher CVD risk in SCZ [27–28]. However, data on risk factors identifiable early in the illness course remain limited. Specifically, findings regarding age at onset (AAO) of the SMD are inconclusive, with reports of increased CVD risk with later AAO [29] and higher CVD frequency with AAO before age 50 years [30] in BD, and no significant association with CVD risk in SCZ [27]. While substance use disorders (SUDs) constitute an independent risk factor for CVD [31], studies of SMD with comorbid SUD are lacking. The same applies to self-harming and suicidal behavior, which is prevalent in SMD [32–34] and related to CVD [35, 36]. Notably, self-harming and lifestyle behavior may be linked to emotional regulatory functions [37–40], which are of importance for CVD risk [41, 42].

Identifying SMD clinical traits associated with CVD development might inform CVD prevention efforts and ultimately improve the long-term health outcomes in individuals with SMD. The primary aim of the current study is to investigate the association between SMD clinical traits with time to CVD onset, defined as an individual’s first recorded CVD diagnosis in national primary and specialist health care registries from 2006 to 2020. We hypothesize that early identifiable clinical traits and related factors, such as disorder onset at a younger age, comorbid SUDs, and self-harming behaviors (SHBs), will be associated with an earlier onset of CVD in individuals with SMD.

## Methods

### Study setting

The present study is a sub-project of the Thematically Organized Psychosis (TOP) study conducted at the University of Oslo and Oslo University Hospital [43, 44]. Study data of healthy controls (HCs, *N* = 1,201) and participants with SMD (*N* = 1,627) recruited from the psychiatric services of the major hospitals in Oslo and the hospitals of Innlandet and Kristiansund, during the period October 2002–September 2019, were linked with national health registry data spanning the period 2006–2020. SMD participants were categorized in the SCZ group (*N* = 998) based on fulfilling the Diagnostic and Statistical Manual of Mental Disorders (DSM)-IV diagnostic criteria for schizophrenia, schizophreniform disorder, schizoaffective disorder, delusional disorder, or psychotic disorder not otherwise specified (NOS). Likewise, participants fulfilling the DSM-IV diagnostic criteria for bipolar I disorder, bipolar II disorder, bipolar disorder NOS, or major depressive disorder with psychotic features [45] were assigned to the BD group (*N* = 629). SMD participants and HCs were between 18 and 65 years of age. Exclusion criteria for SMD participants were severe somatic illnesses potentially interfering with brain functioning, including neurological disorders, a history of severe head injury, and an IQ < 70. Moreover, SMD participants with a known CVD event before the start of the observation period were excluded from the statistical analyses between clinical traits and time to first CVD diagnosis. HCs were randomly selected from the Oslo catchment area using the national population registry of Norway. HCs were interviewed and screened with the Primary Care Evaluation of Mental Disorders [46] to exclude individuals with any current or earlier SMD or depression, those with first-degree relatives with SMD, and individuals with drug abuse or dependency.

All participants gave written informed consent. The study followed the guidelines in the Declaration of Helsinki and was approved by the Regional Committee for Medical Research Ethics (2009/2485).

### CVD data

CVD diagnoses and time of diagnosis were available from the primary health care service from the Norway Control and Payment of Health Reimbursement database (KUHR) for the period 2006–2019, and from the specialist health care service from the Norwegian Patient Registry (NPR) for the period 2008–2020. CVD diagnoses from KUHR and NPR included in the study are given in Supplementary Material 1; all diagnoses from chapter K in the International Classification of Primary Care (ICPC-2), except for marginal conditions (infections, neoplasms, congenital conditions, and insignificant diagnoses) and unspecific arrhythmia, constituting K70 (Infection of circulatory system), K72 (Neoplasm cardiovascular), K73 (Congenital anomaly cardiovascular), K79 (Paroxysmal tachycardia), K80 (Cardiac arrhythmia NOS), K81 (Heart/arterial murmur NOS), K88 (Postural hypotension), K95 (Varicose veins of leg), and K96 (Haemorrhoids), were included. The presence or absence of a CVD diagnosis was coded for each participant (dichotomous variable), and the time of the first occurrence of a CVD diagnosis, as registered in any of KUHR or NPR, was identified.

For merging KUHR and NPR data, the International Classification of Diseases version 10 [47] codes of interest were converted to the following ICPC-2 codes, defining CVD in the current analyses, by using the conversion file “File 4 2023 – Conversion file ICD-10 to ICPC-2” (Excel) at https://www.ehelse.no/kodeverk-terminologi/icpc-2.den-internasjonale-klassifikasjonen-for-primaerhelsetjenesten, from the Directorate of e-health, Norway (from January 2024 merged with the Norwegian Directorate of Health): K71(Rheumatic fever/heart disease), K74 (Ischaemic heart disease with angina), K75 (Acute myocardial infarction), K76 (Ischaemic heart disease without angina), K77 (Heart failure), K78 (Atrial fibrillation/flutter), K82 (Pulmonary heart disease), K83 (Heart valve disease NOS), K84 (Heart disease other), K85 (Elevated blood pressure), K86 (Hypertension uncomplicated), K87 (Hypertension complicated), K89 (Transient cerebral ischaemia), K90 (Stroke/cerebrovascular accident), K91 (Cerebrovascular disease), K92 (Atherosclerosis/peripheral vascular disease), K93 (Pulmonary embolism), K94 (Phlebitis/thrombophlebitis), and K99 (Cardiovascular disease other). From NPR, the main three-character code ICD-10 diagnosis category was utilized, as information on ICD-10 diagnostic subcategories was not regularly given.

### Demographic and clinical data

Demographic data, including information about tobacco use defined as tobacco smoking and/or using snuff (current), were collected by interviews and medical records. Diagnostic evaluations were conducted by physicians and psychologists using the Structured Clinical Interview for DSM-IV Axis I Disorders (SCID-I) [48]. Inter-rater reliability was ensured by scoring a series of videos [49], demonstrating an overall kappa score of 0.92 and 0.99 [50]. We assessed psychotic symptoms with the Positive and Negative Syndrome Scale (PANSS) [51], manic symptoms with the Young Mania Rating Scale [52], and depressive symptoms with the Inventory of Depressive Symptomatology-Clinician Rated [53] (BD group) and the Calgary Depression Scale for Schizophrenia [54] (SCZ group). Clinical information was verified and supplemented using medical records.

### Clinical traits and related factors

*SHB*: As part of the study protocol, SMD participants were assessed for SHB by the question: “Have you ever intentionally overdosed (for example by pills or other medicine) or tried to hurt yourself in any way (like cutting yourself)?” The alternatives for answers were “No” and “Yes”; if “Yes,” the participant was asked to specify the number of times. SHB was categorized into the following three groups: (1) No SHB, (2) SHB one time, and (3) SHB more than once (“recurring”).

*SHB with suicide attempt(s)* (*SHB-SA*): Suicide attempts were assessed after confirmation of SHB, asking how many of those episodes were suicide attempts.

Early TOP study protocols mapped suicide attempts without parallel assessments of SHB; for participants mapped this way (about 12%), the number of SHB and SHB-SA were set equal to each other.

*AAO*: For participants with a schizoaffective disorder, AAO was defined as the age of the first SCID-I-verified illness episode, affective or psychotic, consistent with defining onset as the earliest age at which symptoms caused distress or impaired functioning [55]. For other SCZ diagnoses, AAO was defined as the age of the first SCID-I-verified psychotic episode [56]. For participants with BD, AAO was defined as the age of the first SCID-I-verified affective episode (depressive, hypomanic, manic, or mixed) [57, 58].

*SUD*: Lifetime diagnoses of substance abuse or dependency were based on the SCID-I E-module interview. Dichotomous variables of lifetime SUD (across all substances) versus no lifetime SUD, and specifically, lifetime cannabis use disorder (CUD) versus no lifetime CUD, were made by combining data of abuse and dependency diagnoses. We analyzed CUD specifically based on its associations with SMDs and CVDs [59, 60].

### Statistical analyses

We performed statistical analyses with IBM SPSS Statistics for Windows, Version 27.0–29.0. The level of significance was set at *p* < .05 for all statistical analyses. For sample descriptives, we applied the Kruskal–Wallis test and the Mann–Whitney *U*-test for analyses of non-normally distributed continuous variables, and the chi-square test for categorical variables. We examined normality of data using histograms, *Q*–*Q* plots, and the Kolmogorov–Smirnov test.

The follow-up period started in 2006 and ended at the first occurrence of CVD diagnosis or in 2020, whichever came first. Cox proportional hazards regression was applied to assess hazard ratios of first-time CVD events as registered in KUHR or NPR. We first performed a Cox proportional hazards regression using the total sample (*N* = 2,828) to describe CVD onset in the SMD groups relative to HC, with SCZ diagnosis versus HC and BD diagnosis versus HC as exposures while controlling for age and sex.

In the main analyses, we performed Cox proportional hazards regressions in the SMD participant sample (*N* = 1,627) to examine associations between the clinical traits and related factors (SHB, SHB-SA, AAO, SUD, and CUD) and time to first CVD diagnosis. Age, sex, diagnosis (SCZ and BD), and tobacco smoking [61, 62] were included as covariates in the statistical models.

The proportional hazards assumption of the Cox regressions was tested for the models with significant findings. Adding the interaction between the predictor variable and the time variable yielded a nonsignificant improvement in fit, and none of the interactions were significant (*p* = .315 and *p* = .298). Thus, none of the predictor variables needed to be treated as time-dependent, and the assumption for the Cox proportional hazards model was met.

Sensitivity analyses of the significant findings were performed due to the limited period of registry data (see Supplementary Table 1; CVD/death as outcome). Furthermore, based on associations between cholesterol, impulsivity, and suicide risk [63, 64], we tested the potential impact of serum lipids on the significant findings by including non-high-density lipoprotein (HDL) cholesterol [65] as a covariate in the analyses of SHB more than once and SHB-SA (see Supplementary Table 2). Lastly, to explore potential differences between males and females, we conducted analyses stratified by sex (see Supplementary Tables 3 and 4).

## Results

### Demographic, clinical, and CVD data

There were significantly more males (*p* < .001) in the SCZ group compared to the BD and HC groups. Also, there were significantly more males in the HC group than in the BD group (*p* < .001). Participants were significantly younger in the SCZ group compared to the BD and HC groups (*p* < .001). The median age at inclusion was 28 years in SCZ, and 30 and 33 years in the BD and HC groups, respectively. The number of first-time CVD diagnoses (years 2006–2020) was significantly higher in the BD and SCZ groups compared to the HCs (*p* < .001), and significantly higher in the BD group compared to the SCZ group (*p* < .001). The SCZ group showed significantly higher PANSS total score than the BD group (*p* < .001). AAO was significantly lower in the BD group than in the SCZ group (*p* < .001). See Table 1 for detailed information.

**Table 1.** Sample characteristics

	SCZ (*N* = 998)	BD (*N* = 629)	HC (*N* = 1,201)	*p*-value^e^	Group comparisons
	*N* (%)	*N* (%)	*N* (%)		
Sex, male	592 (59.3)	252 (40.1)	629 (52.4)	<.001	SCZ > HC > BD
Tobacco use (yes)	543 (54.4)	329 (52.3)	-	.293	-
First-time CVD^a^	221 (22.1)	162 (25.8)	227 (18.9)	.003	BD > SCZ > HC
Self-harming (no)	486 (56.5)	295 (55.7)	-	.799	-
One time	144 (14.4)	83 (13.2)	-	.648	-
More than once	230 (23.0)	152 (24.2)	-	.470	-
Suicide attempt(s) (yes)^b^	271 (28.2)	181 (29.7)	-	.822	-
Abuse/dependence (yes)	255 (25.6)	139 (22.1)	-	.128	-
Cannabis (yes)	76 (7.6)	35 (5.6)	-	.134	-
Antipsychotics use (yes)	835 (83.8)	315 (50.1)	-	<.001	SCZ > BD
One AP (only)	615 (61.7)	278 (44.2)	-	<.001	SCZ > BD
Two or more AP	220 (22.1)	37 (5.9)	-	<.001	SCZ > BD
Olanzapine (yes)	318 (31.9)	123 (19.6)	-	<.001	SCZ > BD
Clozapine (yes)	33 (3.3)	1 (.002)	-	<.001	SCZ > BD

	Median (IQR)	Median (IQR)	Median (IQR)		
Age (years)	28.0 (14.0)	30.0 (17.0)	33.0 (15.0)	<.001	BD, HC > SCZ
Self-harming ^w/^suicide attempt(s)^c^	0 (1.0)	0 (1.0)	-	.822	-
PANSS total score	61.0 (22.0)	45.0 (14.0)	-	<.001	SCZ > BD
YMRS score	-	2.0 (4.0)	-		-
IDS-C score^d^	-	15.0 (17.0)	-		-
CDSS score^d^	5.00 (7.0)	-	-	.664	-
Age at onset (years)	22.0 (9.0)	19.0 (9.0)	-	<.001	SCZ > BD
Serum total cholesterol (mmol/L)	5.0	4.9	4.9	<.001	SCZ > BD, HC
Systolic BP (mmHg)	120.0	120.0	-	.019	SCZ > BD
Diastolic BP (mmHg)	77.5	75.0	-	.002	SCZ > BD

Abbreviations: AP, antipsychotic drug(s); BD, bipolar spectrum disorder; BP, blood pressure; CDSS, = Calgary Depression Scale for Schizophrenia; CVD, cardiovascular disease; HC, healthy control; IDS-C, Inventory of Depressive Symptomatology, Clinician-Rated; IQR, interquartile range; PANSS, Positive and Negative Syndrome Scale; SCZ, schizophrenia spectrum disorder; YMRS, Young Mania Rating Scale.

*Note:* Missingness: Tobacco use: 1.6%; Self-harming: 14.8%; Suicide attempt(s): 3.7%; Antipsychotics: 0.1%; Self-harming ^w/^suicide attempt(s): 3.7%; PANSS: 1.9%; YMRS: 2.7%; IDS-C: 6.8%; CDSS: 21.5%; Age at onset: 2.3%; Serum total cholesterol: 13.4%; Systolic BP: 6.3%; Diastolic BP: 6.3%.

a First-time CVD: occurrence of first CVD diagnosis and registered during 2006–2020.

b One or more suicide attempt(s) versus no suicide attempt.

c Self-harming with suicide attempt(s), (continuous), range: SCZ: 0–75; BD: 0–17.

d CDSS cutoff for moderate depression ≥6; IDS-C cutoff for moderate depression ≥22.

e Mann–Whitney *U*-test or Kruskal–Wallis test for variables represented by median (IQR) and chi-square test for comparison of proportions.

### Diagnostic group and first-time CVD in the total sample

**Figure 1.**
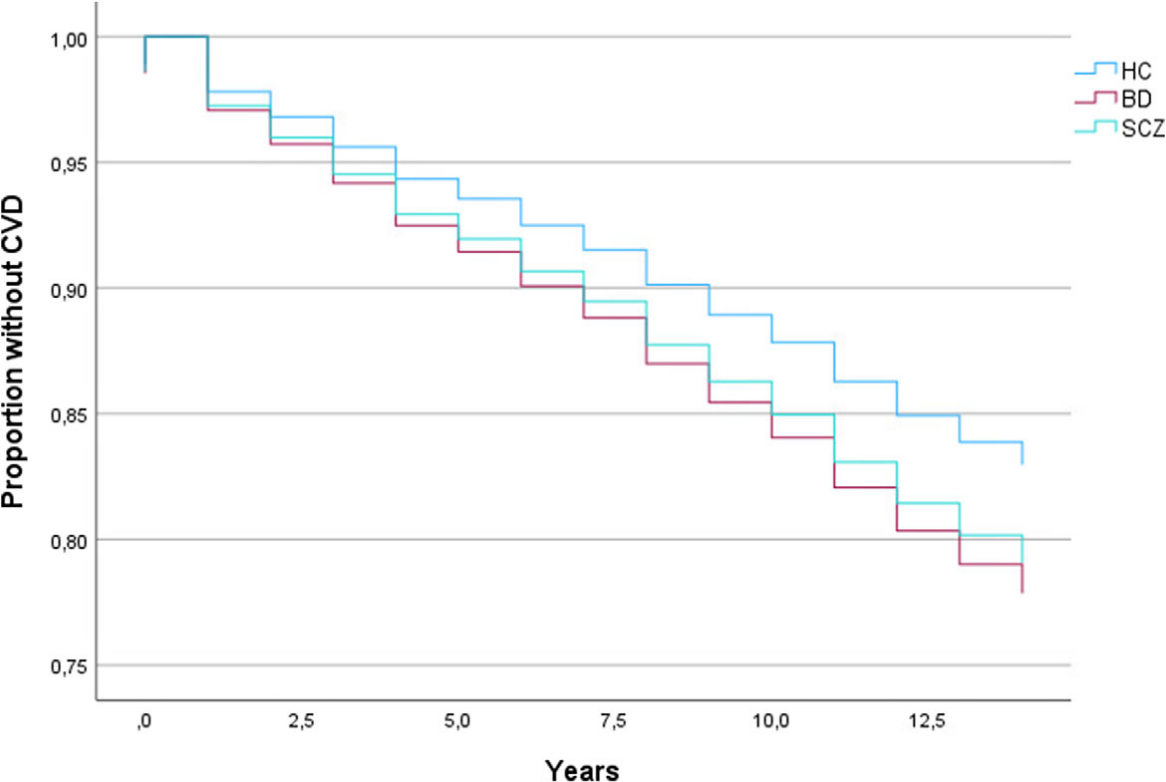
Kaplan–Meier plot showing CVD survival curves according to severe mental disorder diagnostic category and healthy controls. BD, bipolar spectrum disorder (bipolar I disorder, bipolar II disorder, bipolar not otherwise specified, major depressive disorder with psychotic features); CVD, cardiovascular disease; HC, healthy controls; SCZ, schizophrenia spectrum disorder (schizophrenia, schizophreniform disorder, schizoaffective disorder, delusional disorder, psychosis not otherwise specified).

Of all the 2,818 participants (SCZ, BD, and HC), 610 were diagnosed with a first-time CVD (see Table 2). A log-rank test of Kaplan–Meier survival curves of these CVD events revealed significant differences (*p* < .001) between SCZ, BD, and HC (see Figure 1). Multivariate Cox proportional hazards regression models showed a significantly shorter time to the first CVD diagnosis in SCZ and BD relative to HC (*p* = .016 and *p* = .005, respectively; Table 2).

**Table 2.** Cox proportional hazards model of time to first CVD diagnosis in the total sample^a^

	HR (b)	95% CI
Birth year	.951 (−.051)^‡^	.944–.957
Sex	1.211 (.192)*	1.031–1.423
Diagnostic group		
-BD^b^	1.340 (.293)^†^	1.093–1.642
-SCZ^c^	1.257 (.229)*	1.044–1.513

Abbreviations: BD, bipolar spectrum disorder (bipolar I disorder, bipolar II disorder, bipolar disorder not otherwise specified, major depressive disorder with psychotic features); b, beta value; CI, confidence interval; CVD, cardiovascular disease; HR, hazard ratio; HC, healthy control; SCZ, schizophrenia spectrum disorder (schizophrenia disorder, schizophreniform disorder, schizoaffective disorder, other psychosis). Significance: *p < .05; ^†^*p* < .01; ^‡^*p* < .001.

a *N* = 2,818 (610 with first-time CVD; 2,208 right-censored).

b BD versus HC (*p* = .005).

c SCZ versus HC (*p* = .016).

### Clinical and behavior-related traits and first-time CVD in the SMD sample

**Figure 2.**
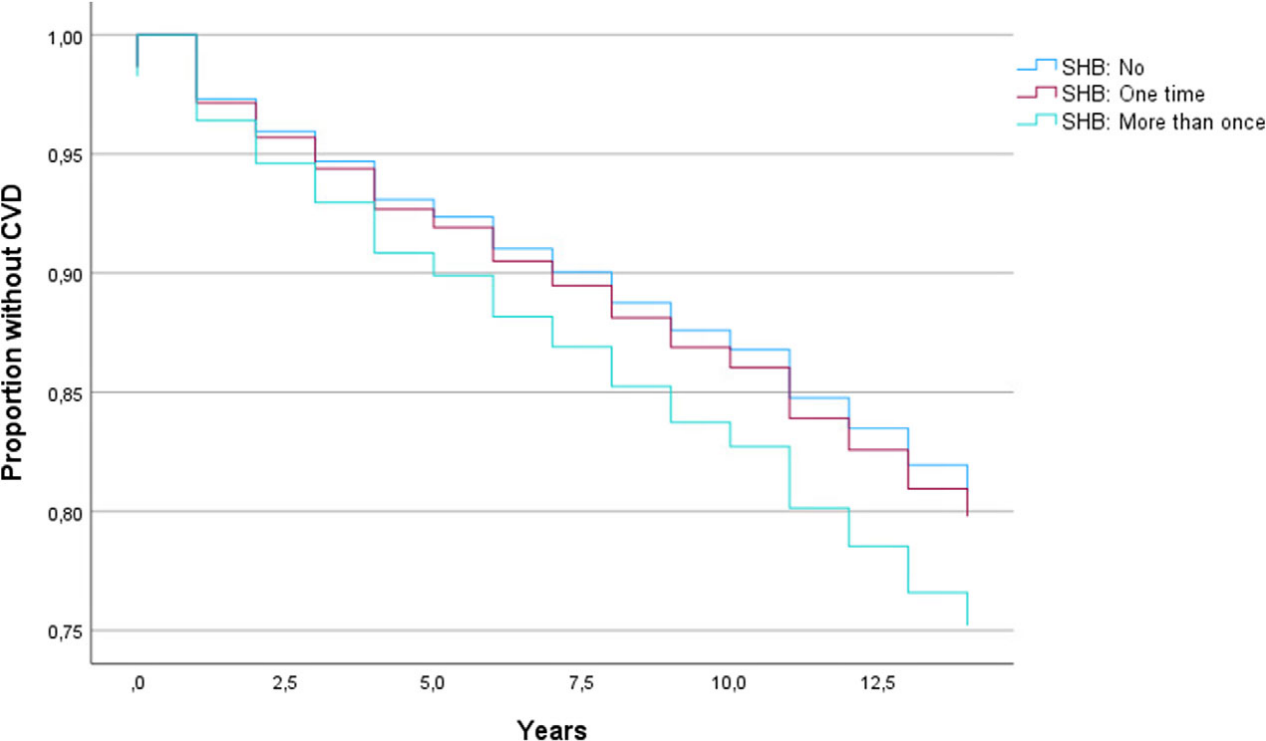
Kaplan–Meier plot showing cardiovascular disease survival curves for different categories of self-harming behavior (SHB). BD, bipolar spectrum disorder (bipolar I disorder, bipolar II disorder, bipolar not otherwise specified, major depressive disorder with psychotic features); CVD, cardiovascular disease; HC, healthy controls; SCZ, schizophrenia spectrum disorder (schizophrenia, schizophreniform disorder, schizoaffective disorder, delusional disorder, psychosis not otherwise specified).

Multivariate Cox proportional hazards regression models showed that SHB was more than once relative to no SHB (Figure 2), was significantly associated with shorter time to first CVD diagnosis in SMD (hazard ratio [HR] = 1.339, *p* = .029; Table 3). A higher number of SHB-SA was associated with a shorter time to first getting a CVD diagnosis (HR = 1.025, *p* = .041, Table 3). Significant results were withheld in sensitivity analyses with time to CVD/death as outcome (see Supplementary Table 1). Non-HDL cholesterol was not a significant covariate in sensitivity analyses of associations of SHB more than once and SHB-SA with time to first CVD diagnosis, and the beta coefficients of SHB more than once and SHB-SA remained in the same order of magnitude (*b* = .339, *p* = .016 and *b* = .023, *p* = .069, respectively; Supplementary Table 2) as in the main analyses. In analyses stratified by sex, beta coefficients in males and females were of a similar order of magnitude for SHB more than once (*b* = .261 and *b* = .268, respectively), but somewhat different for SHB-SA (*b* = .104 and *b* = .017, respectively). Only the association for SHB-SA in males was significant (*p* = .006) (see Supplementary Tables 3 and 4 for details).

**Table 3.** Cox proportional hazards models of time to first CVD diagnosis in SMD sample

	*SHB* ^a^		*SHB-SA* ^b^		*Age at onset* ^c^		*Abuse/dependence* ^d^		*Cannabis* ^e^	
	HR (b)	95% CI	HR (b)	95% CI	HR (b)	95% CI	HR (b)	95% CI	HR (b)	95% CI
Birth year	.955 (−.046)^‡^	.946–.964	.953 (−.049)^‡^	.945–.960	.952 (−.049)^‡^	.943–.961	.954 (−.047)^‡^	.946–.962	.954 (−.047)^‡^	.946–.962
Sex	1.270 (.239)*	1.007–1.601	1.169 (.156)	.947–1.442	1.132 (.124)	.920–1.394	1.164 (.152)	.947–1.432	1.151 (.140)	.937–1.414
Diagnosis^f^	.940 (−.062)	.747–1.184	.923 (−.080)	.746–1.143	.933 (−.069)	.752–1.157	.932 (−.071)	.756–1.148	.934 (−.069)	.758–1.150
Tobacco use^g^	1.073 (.071)	.855–1.348	1.151 (.141)	.934–1.419	1.174 (.160)	.954–1.445	1.168 (.155)	.945–1.443	1.140 (.131)	.928–1.399
SHB										
-One time^h^	1.061 (.059)	.776	–	–	–	–	–	–	–	–
-More than once^i^	1.339 (.292)*	1.031–1.738	–	–	–	–	–	–	–	–
SHB-SA^j^	–	–	1.025 (.025) *	1.001–1.049	–	–	–	–	–	–
Age at onset^k^	–	–	–	–	.997 (−.003)	.985–1.010	–	–	–	–
Abuse/dependence^l^	–	–	–	–	–	–	.894 (−.113)	.696–1.148	–	–
-Cannabis^m^	–	–	–	–	–	–	–	–	.989 (−.011)	.631–1.550

Abbreviations: BD, bipolar spectrum disorder (bipolar I disorder, dipolar II disorder, bipolar disorder not otherwise specified, major depressive disorder with psychotic features); b, beta value; CI, confidence interval; CVD, cardiovascular disease; HR, hazard ratio; SHB, self-harming behavior; SHB-SA, self-harming behavior with suicide attempt; SCZ, schizophrenia spectrum disorder (schizophrenia, schizophreniform disorder, schizoaffective disorder, other psychosis); SMD, severe mental disorder. Significance: *p < .05; ^†^*p* < .01; ^‡^*p* < .001 (exact *p*-values: SHB more than once: *p* = .029, SHB-SA: *p* = .041).

a *N* = 1,385 (314 with first-time CVD; 1,071 right-censored).

b *N* = 1,545 (364 with first-time CVD; 1,181 right-censored).

c *N* = 1,558 (368 with first-time CVD; 1,190 right-censored).

d *N* = 1,597 (377 with first-time CVD: 1,220 right-censored).

e *N* = 1,597 (377 with first-time CVD; 1,220 right-censored).

f SCZ versus BD.

g Currently using tobacco (yes/no).

h SHB one time versus none.

i SHB more than once versus none.

j Number of SHB-SA.

k Age at onset of disorder.

l Diagnosis of drug abuse or dependency (yes/no).

m Cannabis abuse or dependency (yes/no).

There were no significant associations between AAO, SUD, CUD, or diagnosis (SCZ and BD) and time to first CVD diagnosis.

## Discussion

The study demonstrated associations between recurring SHB, a higher number of suicide attempts, and a shorter time to first CVD diagnosis in SMDs. No significant associations were found between AAO, SUDs including cannabis, and CVD onset. Comparisons with HCs confirmed shorter time to CVD onset in SMDs. These findings suggest that SMD clinical traits and related factors may provide illness-specific information relevant to CVD outcomes, beyond standard cardiovascular risk factors.

While the evidence for elevated CVD risk in SMD is robustly established [66], the association with illness-specific factors remains largely unknown. To the best of our knowledge, no previous study has reported associations between SHB and time to subsequent CVD in individuals with SMD. Both the broader self-harming actions and the suicide attempt traits were identified as significantly associated with CVD onset, supporting a link across the spectrum of SHB. The group with recurring self-harming showed a 33.9% increased risk, at any time point, for experiencing a CVD event, compared to the group without self-harming. For the subgroup with a history of suicide attempt(s), the risk increased by 2.5% for each suicide attempt. The magnitude of these risks suggests that information on recurring SHB behavior and suicide attempts may serve a role for clinicians when assessing the risk of CVD in persons with SMDs. Based on the analyses stratified by sex, one might speculate on a stronger association between SHB-SA and time to first CVD event in males than in females. Notably, associations between SHB, suicide attempts, and cardiovascular health have been demonstrated in other groups. In young individuals, Zhong et al. [67] found that a history of suicide attempts in psychiatric inpatients (*N* = 38) and in a community-based sample (*N* = 303) was associated with increased CVD risk compared to individuals from similar groups with suicide ideation only and HCs. Additionally, a link between suicidal ideation and behavior and adverse health outcomes, including death from natural causes such as CVD, has been indicated in longitudinal studies [68, 69]. Given that SHB and suicide attempts are frequently encountered in clinical practice among patients with SMDs, our findings underscore the importance of heightened vigilance regarding CVD risk in these patients.

While the underlying mechanisms of these associations remain speculative, adverse health behaviors, such as emotional eating and smoking, have been linked to self-harming and suicidal behavior [70, 71]. Smoking is a well-established risk factor for CVD [62, 72], while emotional eating has been linked to CVD risk factors, such as hypertension and obesity [73, 74]. A recent genome-wide association study (GWAS) meta-analysis similarly suggested a role of tobacco smoking as well as impulsivity, which is associated with emotional eating [75], in suicide attempt risk [76]. Furthermore, immune activation has been proposed as a shared pathological mechanism in both CVD and SMD [77–79], with emerging evidence linking it to SHB and suicide attempts [80–84].

The other clinical traits and related factors tested were not significantly associated with time to first CVD diagnosis. While evidence supports that individuals with early AAO SMD constitute a subgroup with worse illness outcomes [85–88], our findings do not support early AAO as a marker of shorter time to CVD onset. In comparison, childhood-onset depression relative to adult-onset depression has been linked to an increased risk of serious CVD events [89, 90]. Additionally, studies suggest that the chronicity of mood symptoms impacts vascular mortality in a dose–response manner [91, 92]. However, by largely covarying out duration of illness through age adjustment, our results do not support early AAO as an independent marker of worse CVD outcomes beyond the impact of illness duration. SUDs are common comorbidities in SMDs [93–95] and are generally linked to increased risk of CVD [96, 97]. However, in the current study, neither comorbid CUD nor SUD in general was significantly associated with CVD onset. This contrasts with the PRIMROSE risk model, in which a history of heavy alcohol consumption was a predictor of CVD in SMD [98]. Compared to our study, the extensive data (*N* = 38,824) in the PRIMROSE study was collected from general practitioners and involved older participants, which could possibly explain some of the differences. Our findings suggest that factors beyond a diagnosis of substance abuse or dependency might be important yet underrecognized for assessing CVD risk in the SMD population. Nonetheless, we cannot rule out that participants with substantial substance use not fulfilling the diagnostic criteria may have concealed actual effects of substance abuse and dependency. Lastly, no significant differences in time to CVD onset were observed between SCZ and BD in our SMD sample. This contrasts with some studies indicating a higher CVD risk in SCZ [99–101]; however, findings are mixed [11]. As the current study examines CVD outcomes rather than CVD risk factors, it is plausible that factors other than conventional CVD risk factors are of particular importance for CVD outcomes in SMD. Notably, the representativeness of the sample is supported by the observed shorter time to first CVD diagnosis in SMD compared to HC, in line with findings from large studies and meta-analyses [66, 100, 102, 103].

### Strengths and limitations

Strengths of this study include a large and well-characterized SMD sample with an HC reference group, as well as data from national health registries covering both primary and specialized health care services. While accounting for several confounders, we cannot rule out residual confounding. However, by adjusting for diagnostic group, variations in factors such as psychopharmacological agent use and metabolic parameters between diagnoses were accounted for in analyses of the clinical traits. Despite the rather uniform public health care system in Norway, and the fact that the current lipid levels seem comparable with those from another region of the country [104], we cannot exclude the possibility of regional differences in lipid levels and other CVD risk factors, which might limit the generalizability of the findings. Similarly, the findings may be country-specific, as differences in CVD risk factors have been demonstrated between various parts of the world [105]. Given the observational design of this study, causal inferences cannot be made, and the bidirectionality between mental and somatic health [106] might have influenced the results. Moreover, we do not have the complete lifetime occurrence of CVD diagnoses from the health registries, leaving some uncertainty regarding potentially unidentified CVD events.

## Conclusion

The need for SMD-specific CVD risk prediction models has been indicated [8]. Our findings highlight that clinicians should be especially attentive to CVD risk in patients with recurring SHB and suicide attempts. Identifying such illness-specific risk factors may enhance CVD prevention efforts and lay the ground for targeted interventions for individuals with SMD. Moreover, these identified traits are relevant for early CVD risk detection, supporting timely surveillance and lifestyle modifications that may have significant implications for long-term health outcomes.

## Supporting information

10.1192/j.eurpsy.2025.10106.sm001 Hoffart Lunding et al. supplementary material Hoffart Lunding et al. supplementary material

## Data Availability

The data that support the findings of this study will be made available upon reasonable request.

## Abbreviations

SMD(s): severe mental disorder(s)
SCZs: schizophrenia spectrum disorders
BDs: bipolar spectrum disorders
CVD: cardiovascular disease

## Supplementary material

The supplementary material for this article can be found at http://doi.org/10.1192/j.eurpsy.2025.10106.
