# Supplementary material for: Self-harming behavior linked to earlier onset of cardiovascular disease in severe mental disorders
Source: Eur Psychiatry. 2025 Sep 15;68(1):e143. doi: 10.1192/j.eurpsy.2025.10106 (PMC12538181; doi:10.1192/j.eurpsy.2025.10106)
Supplement: Hoffart Lunding et al. supplementary material [file S0924933825101065sup001.zip › FinalSupplTable2upd150825last.docx]

Supplementary Table 2. Sensitivity analysis – Cox Proportional Hazards Models of time to first CVD diagnosis in SMD sample, including adjustment for non-HDL cholesterol.

|  | *SHB^a^*  HR (b) 95% CI | | *SHB-SA^b^*  HR (b) 95% CI | |
| --- | --- | --- | --- | --- |
| Birthyear | .956 (-.045)‡ | .947-.966 | .953 (-.048)‡ | .944-.962 |
| Sex | 1.269 (.238) | 0.990-1.627 | 1.156 (.145) | .923-1.449 |
| Diagnosis*^c^* | .865 (-.145) | .676-1.107 | .858 (-.153) | .685-1.076 |
| Tobacco use*^d^* | 1.053 (.051) | .826-1.341 | 1.135 (.127) | .909-1.418 |
| Non-HDL cholesterol*^e^* | 1.058 (.057) | .950-1.179 | 1.032 (.032) | .934-1.142 |
| SHB  -One time*^f^*  -More than once*^g^* | 1.170 (.157)  1.404 (.339)^*^ | .847-1.616  1.065-1.850 | -  - | -  - |
| SHB-SA*^h^* | - | - | 1.023 (.023) | .934-1.142 |

*^a^*N=1177 (278 with first-time CVD; 899 right-censored); *^b^*N=1322 (326 with first-time CVD; 996 right-censored); *^c^*SCZ vs. BD; *^d^*Currently using tobacco (yes/no); *^e^*Total cholesterol minus HDL cholesterol; *^f^*SHB one time vs. none; *^g^*SHB more than once vs. none; *^h^*Number of SHB-SA.

Abbreviations: BD, Bipolar Spectrum Disorder (Bipolar I Disorder, Bipolar II Disorder, Bipolar Disorder Not Otherwise Specified, Major Depressive Disorder with Psychotic Features); b, Beta value; CI, Confidence interval; CVD, Cardiovascular disease; HR, Hazard ratio; SHB, Self-harming behavior; SHB-SA, Self-harming behavior with suicide attempt; SCZ, Schizophrenia spectrum disorder (Schizophrenia, Schizophreniform Disorder, Schizoaffective Disorder, Other psychosis); SMD, Severe Mental Disorder. Significance: ^*^p<.05; †p <.01; ‡p <.001 (exact p-values: SHB more than once: p=.016, SHB-SA: p=.069).
