# Supplementary material for: Self-harming behavior linked to earlier onset of cardiovascular disease in severe mental disorders
Source: Eur Psychiatry. 2025 Sep 15;68(1):e143. doi: 10.1192/j.eurpsy.2025.10106 (PMC12538181; doi:10.1192/j.eurpsy.2025.10106)
Supplement: Hoffart Lunding et al. supplementary material [file S0924933825101065sup001.zip › suppl1matonlyfinalfinal.docx]

Supplementary material.

Supplementary material 1. CVD diagnoses included in the study.

K71 Rheumatic fever/heart disease

K74 Ischemic heart disease with angina

K75 Acute myocardial infarction

K76 Ischemic heart disease without angina

K77 Heart failure

K78 Atrial fibrillation/flutter

K82 Pulmonary heart disease

K83 Heart valve disease NOS

K84 Heart disease other

K85 Elevated blood pressure

K86 Hypertension uncomplicated

K87 Hypertension complicated

K89 Transient cerebral ischaemia

K90 Stroke/cerebrovascular accident

K91 Cerebrovascular disease

K92 Atherosclerosis/peripheral vascular disease

K93 Pulmonary embolism

K94 Phlebitis/thrombophlebitis

K99 Cardiovascular disease other
