# Supplementary material for: Self-harming behavior linked to earlier onset of cardiovascular disease in severe mental disorders
Source: Eur Psychiatry. 2025 Sep 15;68(1):e143. doi: 10.1192/j.eurpsy.2025.10106 (PMC12538181; doi:10.1192/j.eurpsy.2025.10106)
Supplement: Hoffart Lunding et al. supplementary material [file S0924933825101065sup001.zip › SupplTable3upd150825last.docx]

|  | *SHB^a^*  HR (b) 95% CI | | *SHB-SA^b^*  HR (b) 95% CI | | *Age at onset^c^*  HR (b) 95% CI | | *Abuse/dependence^d^*  HR (b) 95% CI | | *Cannabis^e^*  HR (b) 95% CI | |
| --- | --- | --- | --- | --- | --- | --- | --- | --- | --- | --- |
| Birthyear | .942 (-.059)‡ | .930-.955 | .945 (-.057)‡ | .933-.956 | .944 (-.058)‡ | .931-.957 | .946 (-.056)‡ | .935-.957 | .946 (-.055)‡ | .935-.958 |
| Diagnosis*^f^* | 1.076 (.073) | .768-1.508 | 1.066 (.064) | .780-1.455 | 1.063 (.061) | .778-1.451 | 1.052 (.051) | .775-1.429 | 1.057 (.056) | .778-1.436 |
| Tobacco use*^g^* | 1.190 (.174) | .864-1.639 | 1.313 (.272) | .977-1.765 | 1.358 (.160)^*^ | 1.010-1.825 | 1.312 (.271) | .971-1.772 | 1.291 (.256) | .964-1.729 |
| SHB  -One time*^h^*  -More than once*^i^* | .856 (-.155)  1.298 (.261) | .547-1.341  .888-1.899 | -  - | -  - | -  - | -  - | -  - | -  - | -  - | -  - |
| SHB-SA*^j^* | - | - | 1.109 (.104)† | 1.030-1.194 | - | - | - | - | - | - |
| Age at onset*^k^* | - | - | - | - | .998 (-.002) | .980-1.017 | - | - | - | - |
| Abuse/dependence*^l^* | - | - | - | - | - | - | .924 (-.079) | .673-1.270 | - | - |
| -Cannabis*^m^* | - | - | - | - | - | - | - | - | .953 (-.048) | .639-1.421 |

Supplementary Table 3. Cox Proportional Hazards Models of time to first CVD diagnosis for males in SMD sample.

*^a^*N=708 (166 with first-time CVD; 542 right-censored); *^b^*N=799 (192 with first-time CVD; 607 right-censored); *^c^*N=802 (192 with first-time CVD; 610 right-censored); *^d^*N=823 (198 with first-time CVD; 625 right-censored); *^e^*N=823 (198 with first-time CVD; 625 right-censored); *^f^*SCZ vs. BD; *^g^*Currently using tobacco (yes/no); *^h^*SHB one time vs. none; *^i^*SHB more than once vs. none; *^j^*Number of SHB-SA; *^k^*Age at onset of disorder; *^l^*Diagnosis of drug abuse or dependency (yes/no); *^m^*Cannabis abuse or dependency (yes/no).

Abbreviations: BD, Bipolar Spectrum Disorder (Bipolar I Disorder, Bipolar II Disorder, Bipolar Disorder Not Otherwise Specified, Major Depressive Disorder with Psychotic Features); b, Beta value; CI, Confidence interval; CVD, Cardiovascular disease; HR, Hazard ratio; SHB, Self-harming behavior; SHB-SA, Self-harming behavior with suicide attempt; SCZ, Schizophrenia spectrum disorder (Schizophrenia, Schizophreniform Disorder, Schizoaffective Disorder, Other psychosis); SMD, Severe Mental Disorder. Significance: ^*^p<.05; †p <.01; ‡p <.001 (exact p-values: SHB more than once: p=.178, SHB-SA: p=.006).
