# Supplementary material for: Self-harming behavior linked to earlier onset of cardiovascular disease in severe mental disorders
Source: Eur Psychiatry. 2025 Sep 15;68(1):e143. doi: 10.1192/j.eurpsy.2025.10106 (PMC12538181; doi:10.1192/j.eurpsy.2025.10106)
Supplement: Hoffart Lunding et al. supplementary material [file S0924933825101065sup001.zip › suppltable4upd150825last.docx]

|  | *SHB^a^*  HR (b) 95% CI | | *SHB-SA^b^*  HR (b) 95% CI | | *Age at onset^c^*  HR (b) 95% CI | | *Abuse/dependence^d^*  HR (b) 95% CI | | *Cannabis^e^*  HR (b) 95% CI | |
| --- | --- | --- | --- | --- | --- | --- | --- | --- | --- | --- |
| Birthyear | .966 (-.035)‡ | .953-.978 | .960 (-.041)‡ | .949-.971 | .959 (-.042)‡ | .946-.972 | .961 (-.040)‡ | .950-.972 | .961 (-.039)‡ | .951-.972 |
| Diagnosis*^f^* | .878 (-.130) | .636-1.213 | .842 (-.172) | .624-1.138 | .876 (-.132) | .643-1.194 | .875 (-.134) | .652-1.173 | .881 (-.126) | .657-1.182 |
| Tobacco use*^g^* | .996 (-.004) | .719-1.381 | 1.016 (.016) | .753-1.372 | 1.035 (.034) | .770-1.392 | 1.066 (.064) | .789-1.440 | 1.065 (.063) | .790-1.436 |
| SHB  -One time*^h^*  -More than once*^i^* | 1.294 (.258)  1.307 (.268) | .830-2.019  .905-1.889 | -  - | -  - | -  - | -  - | -  - | -  - | -  - | -  - |
| SHB-SA*^j^* | - | - | 1.018 (.017) | .987-1.049 | - | - | - | - | - | - |
| Age at onset*^k^* | - | - | - | - | .997 (-.003) | .979-1.015 | - | - | - | - |
| Abuse/dependence*^l^* | - | - | - | - | - | - | .814 (-.206) | .537-1.232 | - | - |
| -Cannabis*^m^* | - | - | - | - | - | - | - | - | .705 (-.349) | .365-1.363 |

Supplementary Table 4. Cox Proportional Hazards Models of time to first CVD diagnosis for females in SMD sample.

*^a^*N=677 (148 with first-time CVD; 529 right-censored); *^b^*N=746 (172 with first-time CVD; 574 right-censored); *^c^*N=756 (176 with first-time CVD; 580 right-censored); *^d^*N=774 (179 with first-time CVD; 595 right-censored); *^e^*N=774 (179 with first-time CVD; 595 right-censored); *^f^*SCZ vs. BD; *^g^*Currently using tobacco (yes/no); *^h^*SHB one time vs. none; *^i^*SHB more than once vs. none; *^j^*Number of SHB-SA; *^k^*Age at onset of disorder; *^l^*Diagnosis of drug abuse or dependency (yes/no); *^m^*Cannabis abuse or dependency (yes/no).

Abbreviations: BD, Bipolar Spectrum Disorder (Bipolar I Disorder, Bipolar II Disorder, Bipolar Disorder Not Otherwise Specified, Major Depressive Disorder with Psychotic Features); b, Beta value; CI, Confidence interval; CVD, Cardiovascular disease; HR, Hazard ratio; SHB, Self-harming behavior; SHB-SA, Self-harming behavior with suicide attempt; SCZ, Schizophrenia spectrum disorder (Schizophrenia, Schizophreniform Disorder, Schizoaffective Disorder, Other psychosis); SMD, Severe Mental Disorder. Significance: ^*^p<.05; †p <.01; ‡p <.001 (exact p-values: SHB more than once: p=.154, SHB-SA: p=.257).
